# Evaluation of transduction efficiency in macrophage colony-stimulating factor differentiated human macrophages using HIV-1 based lentiviral vectors

**DOI:** 10.1186/1472-6750-11-13

**Published:** 2011-01-31

**Authors:** Francisco J Leyva, Joshua J Anzinger, J Philip McCoy, Howard S Kruth

**Affiliations:** 1 Experimental Atherosclerosis Section, National Heart, Lung, and Blood Institute, NIH, Bethesda, MD, USA; 2 Flow Cytometry Core, National Heart, Lung, and Blood Institute, NIH, Bethesda, MD, USA

## Abstract

**Background:**

Monocyte-derived macrophages contribute to atherosclerotic plaque formation. Therefore, manipulating macrophage function could have significant therapeutic value. The objective of this study was to determine transduction efficiency of two HIV-based lentiviral vector configurations as delivery systems for the transduction of primary human blood monocyte-derived macrophages.

**Results:**

Human blood monocytes were transduced using two VSV-G pseudotyped HIV-1 based lentiviral vectors containing EGFP expression driven by either native HIV-LTR (VRX494) or EF1α promoters (VRX1090). Lentiviral vectors were added to cultured macrophages at different times and multiplicities of infection (MOI). Transduction efficiency was assessed using fluorescence microscopy and flow cytometry. Macrophages transduced between 2 and 120 hours after culturing showed the highest transduction efficiency at 2-hours transduction time. Subsequently, cells were transduced 2 hours after culturing at various vector concentrations (MOIs of 5, 10, 25 and 50) to determine the amount of lentiviral vector particles required to maximally transduce human monocyte-derived macrophages. On day 7, all transduced cultures showed EGFP-positive cells by microscopy. Flow cytometric analysis showed with all MOIs a peak shift corresponding to the presence of EGFP-positive cells. For VRX494, transduction efficiency was maximal at an MOI of 25 to 50 and ranged between 58 and 67%. For VRX1090, transduction efficiency was maximal at an MOI of 10 and ranged between 80 and 90%. Thus, transductions performed with VRX1090 showed a higher number of EGFP-positive cells than VRX494.

**Conclusions:**

This report shows that VSV-G pseudotyped HIV-based lentiviral vectors can efficiently transduce human blood monocyte-derived macrophages early during differentiation using low particle numbers that do not interfere with differentiation of monocytes into macrophages.

## Background

Coronary artery disease is the leading cause of death in the United States and other Western societies [1]. Coronary artery disease develops as a consequence of lipid deposition and foam cell formation in the arterial wall leading to the development of atherosclerotic plaques [2]. Atherosclerosis is considered an inflammatory disease in which the major cell types implicated are macrophages, smooth muscle cells, and T lymphocytes [3]. Macrophages are present in virtually every atherosclerotic plaque and have an important role in foam cell and atherosclerotic plaque formation through the uptake and accumulation of cholesterol [4].

Circulating blood monocytes differentiate into macrophages. Monocytes can migrate through the blood vessel wall into surrounding tissue, where they differentiate into macrophages and accumulate in pathological tissue sites in the body. Macrophages produce proinflammatory and proangiogenic mediators, and function as host cells for pathogens. Due to these functions, macrophages can contribute to the initiation and progression of a wide variety of diseases. Therefore, directly manipulating these hematopoietic cell subsets could have significant therapeutic value [5].

Human blood monocyte differentiation into macrophages can be induced in vitro using three different methods: culture in 1) human serum (HS), 2) fetal bovine serum (FBS) with granulocyte-macrophage colony-stimulating factor (GM-CSF) or 3) FBS with macrophage colony-stimulating factor (M-CSF). In our experience, macrophages differentiated with HS or GM-CSF with FBS show similar phenotype and morphology (i.e., round cells resembling fried eggs) [6,7]. However, the M-CSF differentiated monocyte-derived macrophage has a different phenotype characterized by an elongated shaped macrophage with numerous vacuoles corresponding to macropinosomes, and constitutive uptake of low-density lipoprotein (LDL) [8,9].

Gene transfer methods have been attempted to manipulate gene expression in macrophages. Non-viral methods such as calcium phosphate precipitation, electroporation, and liposomal or polyethylenimine transfection are less time-consuming and allow use of a larger number of constructs per experiment than viral methods. However non-viral gene transfer methods are inefficient at transfecting macrophages [5]. Viral systems generally give higher gene transfer efficiency and longer expression time than non-viral systems, but only allow the transfer of small sizes (less than 8 kb) of foreign DNA [10]. Viral methods using adenoviruses have reported high transduction efficiency, but their lack of integration into the host cell genome makes the expression of any transferred gene transient [11-14]. Retroviruses (except lentivirus) require cell division to integrate their DNA into the host genome to remain stably transduced [15]; therefore, the limited proliferative nature of primary human macrophages does not favor the use of this viral vector.

Lentiviruses like HIV have the capacity to infect non-dividing and dividing cells and to integrate into the host cell genome [16-20]. Due to these characteristics, HIV-based lentiviral vectors have been proposed as good delivery system candidates for gene therapy, but the attempt to use them in clinical trials has raised concerns about their safety including the risk of genetic recombination leading to the generation of replication-competent retrovirus in humans. Further modifications in the packaging and genetic components of viral genes have been carried out to develop safer HIV-based lentiviral vector systems [21-24]. Due to our interest in gene manipulation of human M-CSF differentiated monocyte-derived macrophages, the objective of this study was to determine transduction efficiency of primary human blood M-CSF differentiated monocyte-derived macrophages transduced with two VSV-G pseudotyped HIV-1 based lentiviral delivery systems.

## Results

### Determination of optimal time to transduce

**Figure 1:**
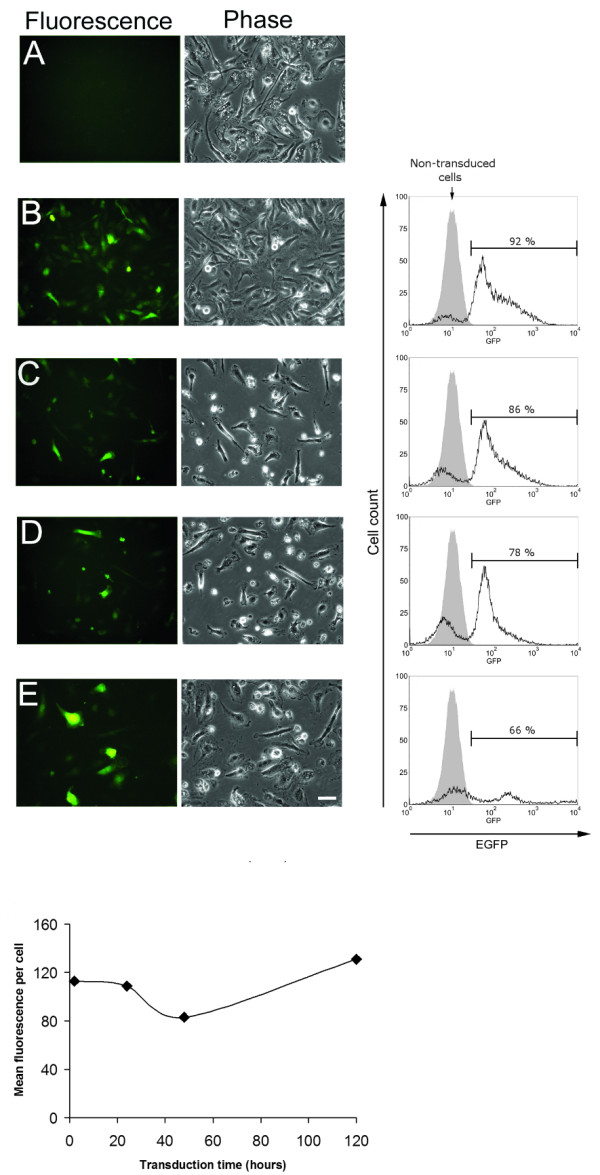
**EGFP expression and flow cytometry analysis of human blood monocyte-derived macrophages transduced at different times**. Cells from donor 1 were transduced with lentivirus VRX1090 using an MOI of 50 (30 × 10^6 ^transducing units/ml) at 2 (B), 24 (C), 48 (D) and 120 (E) hours after cell plating. Control non-transduced cells are shown in A. Each row of the upper panel corresponds to the same microscopic field and respective flow cytometry histogram. Photomicrographs were taken on day 8 after cell plating. Scale bar = 50 μm and applies to all. Each bracketed region in the histogram corresponds to % EGFP-positive cells and shaded area corresponds to non-transduced cell control. Flow cytometry was performed on day 9 after cell plating. Shown in lower panel is mean fluorescence intensity per EGFP-positive cell at different times after cell plating.

Macrophage cultures from the same donor (donor 1) were transduced with VRX1090 vector using an MOI of 50 (final concentration of 30 × 10^6 ^transducing units per ml, TU/ml) at 2, 24, 48 and 120 hours after plating. On day 8, all cultures transduced with VRX1090 showed EGFP-positive cells by microscopic examination (Figure 1A). In addition, cell density assessed by phase microscopy was slightly decreased in cultures transduced at 24, 48 and 120 hours compared with 2 hours after plating. Flow cytometric analysis carried out on day 9 showed a peak shift in data histograms corresponding to the presence of EGFP-positive cells for all transduced cultures compared with non-transduced control cells (Figure 1A). These findings correlated with the qualitative results found by direct visualization of the cells with the fluorescence microscope. All transductions were highly efficient with values between 66 and 92% of cells showing EGFP fluorescence. There was a negative correlation (r = -0.99, p = 0.013) between time of transduction and % EGFP-positive cells.

The results showed that the highest transduction efficiency was reached when monocytes were transduced 2 hours after plating. On the other hand, the amount of fluorescence intensity per cell did not correlate (r = 0.46, p = 0.54) with time of transduction (Figure 1B). Considering these findings, in order to achieve the highest transduction efficiency, all following experiments were performed 2 hours after monocyte plating.

### Determination of optimal multiplicity of infection

**Figure 2:**
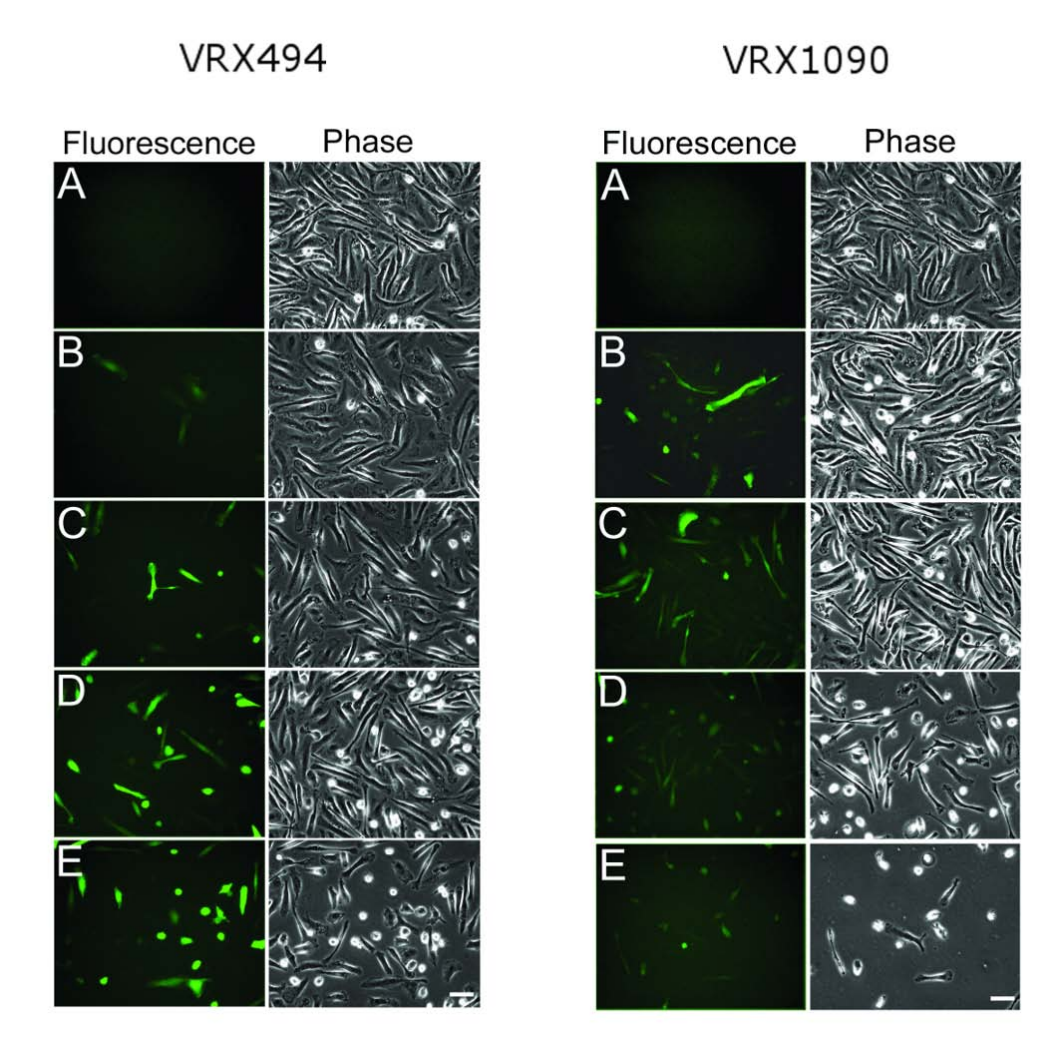
**EGFP expression in human blood monocyte-derived macrophages transduced with VRX494 and VRX1090 at different MOIs**. Cells from donor 2 were transduced with lentivirus 2 hours after plating using MOIs of 5 (B), 10 (C), 25 (D), and 50 (E) at a final concentration of 3, 6, 15, and 30 × 10^6 ^transducing units/ml, respectively. Control non-transduced cells are shown in A. Each row corresponds to the same microscopy field. Photomicrographs were taken on day 7 after cell plating. Scale bar = 50 μm and applies to all.

**Figure 3:**
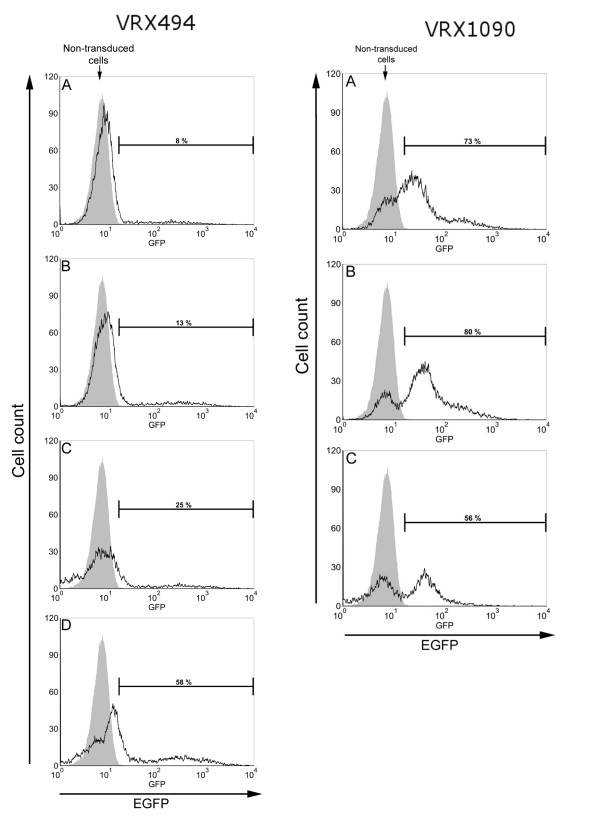
**Flow cytometry analysis of human blood monocyte-derived macrophages transduced with lentivirus at different MOIs**. Cells from donor 2 were transduced with lentivirus VRX494 and VRX1090 two hours after plating using MOIs of 5 (A), 10 (B), 25 (C), and 50 (D) at a final concentration of 3, 6, 15, and 30 × 10^6 ^transducing units/ml, respectively. Each bracketed region in the histogram corresponds to % EGFP-positive cells and shaded area corresponds to non-transduced cell control. Flow cytometry was performed on day 8 after cell plating.

**Figure 4:**
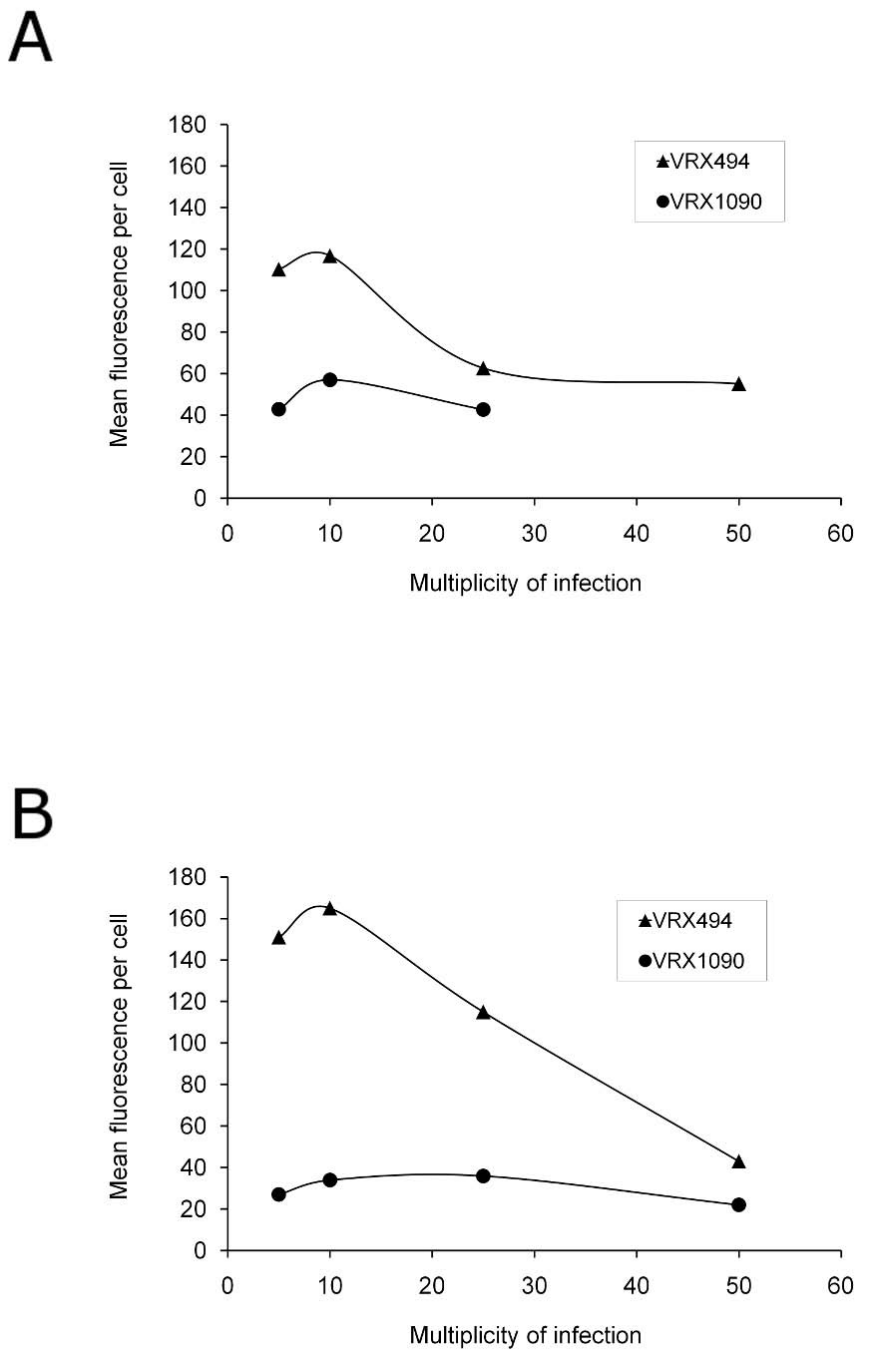
**Transduction efficiency of human blood monocyte-derived macrophages transduced with VRX494 and VRX1090 at different MOIs**. Transduction efficiency as represented by mean fluorescence intensity per EGFP-positive cell of human blood monocyte-derived macrophages from donor 2 (A) and donor 3 (B) transduced with lentivirus VRX494 and VRX1090 at different MOIs. Flow cytometry was performed on day 8 after cell plating.

We next determined the MOI required during transduction to efficiently transduce human monocyte-derived macrophages. In the experiment shown in figures 2, 3 and 4A, macrophage cultures from donor 2 were transduced once at the optimal transduction time, determined above to be 2 hours after plating. Transduction was performed using two different lentivirus vectors VRX494 and VRX1090. Each transduction was carried out using different MOIs: 5, 10, 25, and 50 at a final concentration of 3, 6, 15, and 30 × 10^6 ^TU/ml, respectively.

On day 7, all VRX494-transduced cultures showed EGFP-positive cells (Figure 2). On day 8, % EGFP-positive cells and fluorescence intensity were quantified using flow cytometry. In all transduced cultures a peak shift corresponding to the presence of EGFP-positive cells was found (Figure 3). Transduction efficiency ranged between 8 and 58%, and a positive correlation (r = 0.99, p = 0.007) between MOI and % EGFP-positive cells was found. However, the fluorescence intensity per cell showed a trend toward lower values with higher MOIs used for transduction (Figure 4A).

Photomicrographs of cultures transduced with VRX1090 showed EGFP-positive cells in all conditions (Figure 2). However, at an MOI of 25 and 50 a decrease in cell density compared to non-transduced control was observed by phase microscopy. On day 8, flow cytometry analysis was performed. Since the cell density of the culture transduced with an MOI of 50 was very low, there were insufficient cells for flow cytometry analysis. In all conditions, flow cytometry analysis histograms showed a peak shift for transduced cells when compared with non-transduced cells (Figure 3). All transductions were highly efficient with a range between 56 and 80% EGFP-positive cells. However, % EGFP and mean fluorescence intensity per cell showed no correlation with the MOIs used for transduction (Figures 3 and 4A).

To evaluate the role of the donor in the variability of these results, a second experiment using VRX1090 and VRX494 and different MOIs was repeated using blood monocytes from a different donor (donor 3). As shown in Table 1 and Figure 4B, the results obtained for each vector such as % EGFP-positive cells and mean fluorescence intensity per cell trends were generally similar to the first experiment with donor 2. Also, vector copy numbers per cell were determined in this experiment and generally correlated with % EGFP-positive cells (Table 1). Cultures transduced at MOIs of 25 and 50 showed low cell density, and following flow cytometry there were not enough cells remaining for determination of vector copy numbers.

**Table 1:** Transduction efficiency of human blood monocyte-derived macrophages using different MOIs

MOI	% EGFP-positive cells	G-tag copies per cell
VRX494		
5	23	9
10	39	18
25	67	nd*
50	56	nd
		
VRX1090		
5	83	37
10	90	55
25	74	nd
50	44	nd

*nd = not determined due to low cell number Human blood monocyte-derived macrophages for this experiment were obtained from donor 3

## Discussion

Primary cells are usually more difficult to transfect or transduce than their similar immortalized cell lines [25], and macrophages are one of the most resistant cell types to gene transfer [10]. Therefore, obtaining efficient gene transfer in primary human blood monocyte-derived macrophages could be challenging.

In this report, we found that the best time to transduce human blood monocytes was 2 hours after cell plating. There were progressively fewer EGFP-positive cells when macrophage cultures were transduced at later times following plating of the cells. The differences between transduction time and time of flow cytometry quantification probably do not explain the negative correlation found between time of transduction and % EGFP positive cells. EGFP is a stable protein with a turnover of approximately 10% in 3 hours, whose expression can be detected 24-48 hours after its cDNA has been transferred into various mammalian cell types [26]. Since all transductions in our cell cultures were performed between 72 hours and 192 hours before quantification, it is reasonable to conclude that by the time of flow cytometry quantification, all transduced cultures should have already reached their plateau of EGFP concentration. Thus, the difference observed between each group was likely due to differences in transduction efficiency rather than due to the differences in time of flow cytometry quantification. These findings suggest that monocyte-derived macrophages during the early phases of differentiation are highly transducible with VRX1090, but later they become less susceptible to transduction; the less differentiated the higher the transduction efficiency. However, we cannot exclude the possibility that more differentiated macrophages transduced for longer times would produce higher transduction efficiencies. In any case, transduction at later times after cell plating was associated with lower macrophage cell density, another factor favoring transduction at 2 hours after plating as the optimal time for transduction.

The possibility that macrophages phagocytosed EGFP derived from lentiviral solution contaminated with EGFP expressed during generation of the lentivirus from infected producer cells (i.e., pseudotransduction) cannot explain these findings. In such a case, the results would have been the opposite. In pseudotransduction, cells initially take up EGFP but then metabolize it without producing new EGFP. Thus, in pseudotransduction the cultures that were transduced later should have shown a higher proportion of EGFP-positive cells. Since this is the opposite of the pattern observed in this experiment, pseudotransduction was probably minimal.

Cultures transduced 24, 48 and 120 hour after plating showed a decrease in cell density when compared with the non-transduced control cultures transduced at 2-hours after plating. Since all cultures transduced 24 hours after plating were in the process of macrophage differentiation [27], it is possible that lentivirus addition in these cultures decreased cell proliferation or affected the differentiation of monocytes into macrophages to some degree thereby indirectly decreasing macrophage survival. The possibility of direct lentivirus-induced cell toxicity is remote, because it is well established that cell-induced toxicity correlates with exposure time. In our experiments, the culture with the longest exposure time to the lentivirus was the culture transduced 2 hours after plating. This culture showed the highest cell density compared with the other transduced cultures, so direct lentivirus-induced cell toxicity cannot explain the decrease in cell density. Mean fluorescence intensity per cell did not correlate with transduction time, suggesting that the amount of EGFP expressed per transduced cells is independent of transduction and macrophage differentiation time.

Our findings show that both lentiviruses at any of the various concentrations used were able to transduce monocyte-derived macrophages and produce EGFP in amounts sufficient for detection. Results of transducing monocyte-derived macrophages with VRX494 were similar to those observed in previous reports using viral transducing systems, higher MOIs resulted in higher frequency of cells that were transduced [20,28,29]. Transductions performed with VRX1090 showed a higher frequency of transduced cells than VRX494, but cells transduced with VRX494 showed higher fluorescence intensity per cell. These differences could be explained mostly by macrophage response during transduction (e.g., activation, promoter expression). Since each lentiviral vector used in this study requires a different promoter for protein expression, it is possible that differences in promoter and cell activity could have been responsible of the differences in the levels of EGFP expression. There is less possibility that intrinsic differences between the 2 lentiviruses (e.g., particle size, stability) could explain the differences found between both vectors since the same system was used to generate both lentiviruses.

Previous publications evaluating different viral systems for gene transfer in primary human monocyte-derived macrophages used the three methods previously mentioned to induce cell differentiation: HS, M-CSF and GM-CSF [13,30,31]. Table 2 shows the transduction efficiency (measured as frequency of positive cells), its quantification technique, viral system used, MOI, and blood monocyte-derived macrophage differentiation method reported by each publication [12-14,19-21,28-34]. Only our study using HIV-derived lentivirus reached higher values comparable to those studies in Table 2 using adenovirus viral systems that required higher MOIs and as discussed above without viral DNA incorporation into the host cell genome which is required for stable transduction. However, when comparing our study with previous studies listed in Table 2 where HIV-derived lentivirus was used to transduce human macrophages [19-21,28,29,32,34], only in our study was M-CSF used to induce macrophage differentiation. M-CSF-differentiated macrophages have been reported to show higher transduction efficiency than GM-CSF-differentiated macrophages when adenovirus viral systems are used [30]. This last finding is expected since adenovirus requires integrin α_v_β_5 _for internalization [35], and M-CSF-differentiated macrophages express higher levels of integrin α_v_β_5 _than GM-CSF-differentiated macrophages [30]. VSV-G pseudotyped lentiviral vectors, such as the ones used in this report, have very broad tropism for cell infection [36]. However, it is possible that the transduction efficiency differences observed when comparing M-CSF with GM-CSF-differentiated macrophages may be due to differences in mechanisms available for uptake of VSV-G pseudotyped lentivirus by these two types of macrophages. In this regard, M-CSF differentiated human macrophages show very high levels of constitutive fluid-phase pinocytosis mediated by both macropinocytosis and micropinocytosis [8,9]. Fluid-phase pinocytosis (a non-receptor uptake mechanism) has been shown to mediate enhanced cellular transduction by vesicular stomatitis virus pseudotyped vectors as well as uptake of HIV by M-CSF differentiated human macrophages [37,38].

**Table 2:** Transduction efficiency* of human blood monocyte-derived macrophages using different viral systems

	Transduction efficiency	MOI	Viral system	Differentiation Method	Quantification technique
					
Haddada H, et al. [13]	40-80%	100	Adenovirus	HS	Beta-gal activity
Huang S, et al. [30]	52%	1000	Adenovirus	M-CSF+FBS	Beta-gal activity
Schneider SD, et al. [33]	20%	100	Adenovirus	HS	LacZ activity
De S, et al. [31]	10%	100	Adenovirus	GM-CSF+FBS	Beta-gal activity
Foxwell B, et al. [12]	> 90%	100	Adenovirus	M-CSF+FBS	Beta-gal activity flow cytometry
Heider H, et al. [14]	80%	30	Adenovirus	HS	GFP fluorescence microscopy
White S, et al. [34]	15%	1	HIV/SIV based lentivirus	GM-CSF+HS	GFP fluorescence microscopy
Schroers R, et al. [20]	30%	100	HIV based lentivirus	GM-CSF+FBS	YFP flow cytometry
Neil S, et al. [19]	20%	10	HIV based lentivirus	HS	GFP flow cytometry
Ravot E, et al. [32]	40%	0.65	HIV based lentivirus	HS+FBS	GFP flow cytometry
Lu Y, et al. [21]	40%	50	HIV based lentivirus	HS+FBS	GFP fluorescence microscopy
Jarrosson-Wuilleme L, et al. [28]	30%	25	HIV based lentivirus	GM-CSF+FBS	GFP flow cytometry
Zeng L, et al. [29]	50%	100	HIV based lentivirus	HS+FBS	GFP fluorescence microscopy
Current report	80-90%	10	HIV based lentivirus	M-CSF+FBS	EGFP flow cytometry

* Percentage transduced cells, values correspond to the highest value listed in the respective manuscript. MOI = multiplicity of infection, HS = human serum, GM-CSF = granulocyte-macrophage colony-stimulating factor, M-CSF = macrophage colony-stimulating factor, FBS = fetal bovine serum

Our finding that the optimal time to transduce M-CSF differentiated macrophages was better when these macrophages were less differentiated contrasts with the previous findings reported by Zeng et al. [29] in which less differentiated macrophages were more resistant to transduction. However, Zeng et al. reported transducing macrophages following differentiation with human serum, which produces the GM-CSF macrophage phenotype in contrast to the M-CSF macrophage phenotype we have studied here. Zeng et al. also transduced the macrophages in the presence of polybrene. We did not use polybrene in our experiments because we observed that polybrene was toxic to human M-CSF differentiated monocyte-derived macrophages (unpublished observation).

## Conclusions

If higher MOIs are excluded, where donor-dependent a cell density decrease was usually observed, our study shows that transducing M-CSF differentiated monocyte-derived macrophages with VRX1090 lentivirus using an MOI of 10 consistently gives 80-90% transduction efficiency with no observed decrease in cell density. Thus, we show for first time that HIV-based lentiviruses can efficiently transduce M-CSF differentiated human blood monocyte-derived macrophages early during differentiation using a low MOI that will not interfere with the cell differentiation process or induce a decrease in cell density.

## Methods

### Cell Culture

Human blood monocytes were collected from three healthy blood donors (donor 1, 2 and 3) and purified with counterflow centrifugal elutriation of mononuclear cells [39]. Human blood cell collections were done by the Department of Transfusion Medicine, Clinical Center, National Institutes of Health, under a human subjects research protocol approved by a National Institutes of Health institutional review board and in agreement with the Helsinki Declaration. Cells were plated at 2 × 10^5 ^cells per cm^2 ^in 6-well CellBIND plates (Corning, Corning, NY), and cultured in RPMI 1640 medium (Mediatech, Herndon, VA) with 10% FBS (Invitrogen, Carlsbad, CA). After 2-hours in a cell culture incubator with 5% CO_2_/95% air at 37ºC, cells were rinsed 3 times with RPMI 1640 medium and then cultured in 3 ml RPMI 1640 medium with 10% FBS, 50 ng/ml M-CSF, and 25 ng/ml IL-10, both of the latter obtained from PeproTech (Rocky Hill, NJ). IL-10 was used to induce differentiation of monocytes into macrophages, since IL-10 used together with M-CSF increases M-CSF receptor expression which enhances the macrophage differentiation process [27]. Cell cultures were fed with fresh medium after five days.

### Vector production

Two vesicular stomatitis virus G protein (VSV-G) pseudotyped HIV-1 based lentiviral vectors encoding the enhanced green fluorescent protein (EGFP) driven by either the native HIV LTR promoter (VRX494, derived from NL4-3, Clade B) or the constitutive EF1α promoter in a SIN configuration (VRX1090), both produced by VIRxSYS Corporation (Gaithersburg, MD) were used for these studies. Vectors were produced as previously described [40,41]. In brief, HEK293 cells were transfected using calcium phosphate with the VIRPAC packaging construct, a single packaging plasmid that co-expresses Gag-Pol, Tat, Rev, and VSV-G. Supernatant containing lentiviral particles were collected every 12 h from 24 to 48 h after transfection, concentrated by high-speed centrifugation at 10,000 g for 12 h, and titered by Q-PCR (determination of number of transducing or infectious units per ml) on HeLa-tat cells. Titers for VRX494 and VRX1090 were 3 × 10^9 ^TU/ml (p24 = 47 ug/ml) and 3.9 × 10^9 ^TU/ml (p24 = 92 ug/ml) respectively.

### Transduction of macrophages

Cultured monocyte-derived macrophages were transduced separately with lentiviral vectors VRX494 and VRX1090. Lentiviral vectors were added directly into each well containing 3 ml of culture medium at different Hela-tat based multiplicities of infection (MOI-defined as the number of lentiviral particles able to transduce used per Hela-tat cell). Macrophage transduction efficiency, defined as percentage of transduced cells expressing EGFP and mean fluorescence intensity, was assessed qualitatively using an Olympus IX81 fluorescence microscope (Center Valley, PA) equipped with an FITC filter, and quantitatively using a FACSCalibur flow cytometer (Becton Dickinson Biosciences, San Jose, CA). Because a cell suspension was required for flow cytometry analysis, cells were scraped from each well after a 5-minute incubation with Cellstripper solution (Mediatech), a non-enzymatic cell dissociation mixture of chelators. Non-transduced cells serving as controls also were analyzed with the cytometer. Gating was set on light scatter to include cells while excluding debris. EGFP fluorescence was measured using the 525 nm bandpass channel. EGFP positive cells were defined as those cells having fluorescence intensity greater than the non-transduced control cells. The mean geometric fluorescence intensity per EGFP-positive cell was determined. Data were analyzed using FCS Express version 3 (De Novo Software, Los Angeles, CA).

### Determination of vector copy number per cell

Transduced monocyte-derived macrophages were scraped from each well after a 5-minute incubation with Cellstripper solution (Mediatech). Total genomic DNA was extracted and purified using a QIAamp DNA Micro Kit from Qiagen (Valencia, CA). Vector-specific real-time PCR analysis using primers and probes specific for GFP cDNA (G-tag) were carried out to determine the number of integrated viral genomes. Cell numbers were estimated by measuring the number of β-globin DNA copies per sample by real-time PCR as previously described [42].

### Statistical Analysis

Data was analyzed using Pearson’s correlation coefficient. Statistical analyses were performed using Prism 4 for Windows version 4.00 (GraphPad Software, Inc., La Jolla, CA). Alpha error was set at p < 0.05.

## Competing interests

The authors declare that they have no competing interests.

## Authors' contributions

FJL carried out monocyte cell culture, macrophage differentiation, lentiviral transduction, fluorescence microscope analysis, cell harvesting for EGFP quantification, statistical analysis, and drafted the manuscript. JJA also carried out monocyte cell culture, macrophage differentiation, and fluorescence microscope analysis. JPM provided technical advice, carried out flow cytometry analysis and data interpretation. HSK conceived the study, participated in its design and coordination, and helped to draft the manuscript. All authors read and approved the manuscript.
